# Microbiological assessment reveals that *Salmonella*, *Shigella* and *Campylobacter* infections are widespread in HIV infected and uninfected patients with diarrhea in Mozambique

**DOI:** 10.1371/journal.pgph.0001877

**Published:** 2023-05-22

**Authors:** Delfina Fernandes Hlashwayo, Emília Virgínia Noormahomed, Leonilde Bahule, Constance Benson, Robert T. Schooley, Betuel Sigaúque, Kim E. Barrett, Custódio Gabriel Bila

**Affiliations:** 1 Department of Biological Sciences, Faculty of Sciences, Universidade Eduardo Mondlane, Maputo, MZ; 2 Department of Animal Health & Epidemiology, Faculty of Veterinary Medicine, Universidade Eduardo Mondlane, Maputo, MZ; 3 Department of Microbiology, Faculty of Medicine, Universidade Eduardo Mondlane, Maputo, MZ; 4 Division of Infectious Diseases, Department of Medicine, University of California, San Diego (UCSD), La Jolla, CA, United States of America; 5 Mozambique Institute for Health Education and Research (MIHER), Maputo, MZ; 6 Manhiça Health Research Center (CISM), Manhiça, MZ; 7 Department of Physiology and Membrane Biology, University of California Davis School of Medicine, Sacramento, CA, United States of America; University of Oxford, UNITED KINGDOM

## Abstract

Diarrhea is an important cause of hospitalizations in Mozambique. However, little attention has been paid to the impact HIV infection on the prevalence or clinical manifestations of enteric bacterial infections. This study aimed to determine the prevalence of *Salmonella* spp., *Shigella* spp. and *Campylobacter* spp. in HIV-infected and HIV-uninfected patients with diarrhea, identify risk factors for infection, and explore the association between HIV status, viral load, and bacterial prevalence. We conducted a case-control study at the Centro de Saúde de Mavalane and Centro de Saúde 1° de Maio in Maputo, Mozambique, from November 2021 to May 2022. We recruited 300 patients, including 150 HIV-infected (cases) and 150 HIV-uninfected patients (controls), aged between 0–88 years, presenting with diarrhea. Stool samples were collected for bacterial isolation through culture, and for each HIV-infected patient, 4 ml of venous blood were obtained for viral load detection through PCR. A total of 129 patients (43.0%) had at least one bacterial infection. The prevalence of *Salmonella* spp., *Shigella* spp. and *Campylobacter* spp. was 33.0% (n = 99), 15.0% (n = 45) and 4.3% (n = 13), respectively. The prevalence of any bacterial infection did not differ significantly between HIV-infected (45.3%, n = 68) and HIV-uninfected patients (40.7%, = 61) (p = 0.414). Overall, having 2–3 symptoms of enteric disease (p = 0.008) and a basic education (p = 0.030) were factors associated with bacterial infection. Of the 148 patients for whom HIV-1 RNA levels were available, 115 had copy numbers ≤ 75. Another 13 had levels between 76 and 1,000 and the remaining 20 had an average of 327,218.45 copies/ml. Bivariate logistic regression found that *Shigella* spp. were associated with HIV (p = 0.038), although no association was found in the multivariate analysis. Enteric infections are common in both HIV-infected and -uninfected patients. Low schooling influences the occurrence of enteric infections, which highlights the need to raise awareness about their prevention.

## Introduction

Diarrhea is a major global health issue, ranking as the ninth cause of mortality worldwide [1] and the second leading cause of death in children under the age of five years of age, according to the World Health Organization (WHO) [2]. In 2019, diarrhea resulted in the deaths of over 1,5 million people [1]. In low- and middle-income countries, including those in sub-Saharan Africa, diarrhea accounted for 88% of deaths of children under five years in 2000 [3]. Mozambique is no exception, where diarrhea is an important cause of hospitalizations and death [4]. In fact, it was the eighth-leading cause of death in the country in 2019 [1], and in 2020, there were 514,136 cases of diarrhea and 144 related deaths reported to the national health system [5].

Most cases of acute diarrhea are caused by infectious agents such as viruses, bacteria, and parasites [6], which are typically transmitted via contaminated food and water [2]. Among the bacterial pathogens commonly associated with diarrhea are *Salmonella*, *Shigella*, enterotoxigenic *Escherichia coli* (ETEC), and *Campylobacter* spp. [7]. Several of these pathogens have been found in stools of patients with diarrhea in Mozambique [4]. However, most investigations into enteric pathogens associated with diarrhea in Mozambique have been limited to Maputo Province [8–15], Maputo City [16–18] and selected hospitals throughout the country, including two in the northern region and one in the central region [19, 20]. In these studies, a number of bacteria were detected, including *E*. *coli* [13, 16] and its various pathotypes [8, 11, 12, 14, 16–19], *Shigella* spp. [8, 9, 11, 13, 15, 18], *Salmonella* spp. [8, 10, 18], including non-typhoidal *Salmonella* [11, 13, 15], *Campylobacter* spp. [8, 11, 13, 15, 18], *Helicobacter pylori* [13], *Vibrio* spp. [8, 20] (including *V*. *cholerae* [11] and *V*. *parahaemolyticus* [20]), and other bacteria [11, 18]. Some of these pathogens have been linked to diarrhea, including enterotoxigenic *E*. *coli* and *Shigella* spp. [11]. In one study, enteropathogenic *E*. *coli* was linked to an increased risk of death [13].

To date, most studies on diarrhea in Mozambique have focused on children under the age of five [8, 10–15, 18]. Although this age group is particularly vulnerable, it is essential to expand research efforts to other age groups to gain a comprehensive understanding of the affected population, their associated risk factors, symptoms, and etiologies.

Mozambique is challenged by several diseases that are leading causes of mortality, including HIV/AIDS (25.1%), respiratory infections and tuberculosis (13.8%), neglected tropical diseases and malaria (8.0%), stroke (6.0%), enteric infections (3.9%), among other infectious and non-communicable diseases [1]. Despite the country’s high prevalence of HIV (13.2% among people aged 15–49 years) [21], only one study has explored the association between HIV infection and diarrheal disease [12]. This represents an important gap in the knowledge of diarrhea in Mozambique, as it is unknown whether HIV-infected patients are disproportionately affected, or whether plasma HIV-1 RNA level influences the prevalence or clinical impact of enteric infections. Therefore, the current study aimed to address this gap by defining the prevalence of *Salmonella* spp., *Shigella* spp. and *Campylobacter* spp. in stool samples from HIV infected and HIV uninfected patients with diarrhea from two health units in Maputo, Mozambique. Our study also investigated associated risk factors and sought to evaluate the impact of HIV serostatus, defined by the HIV-1 viral load on the prevalence of the bacteria. By doing so, we aimed to provide new insights into the epidemiology of diarrheal disease in Mozambique.

## Materials and methods

### Study period, area and design

Between November 2021 and May 2022, we conducted a case-control study at the Centro de Saúde de Mavalane and the Centro de Saúde 1° de Maio, which are two healthcare units located in Maputo’s peri-urban and suburban areas.

Maputo city is both capital and the largest city of Mozambique. It is also the main financial, corporate, and commercial center of the country. Maputo city has approximately 1.12 million inhabitants, half of whom reside in unplanned settlements, resulting in a high population density, and poor access to basic safe drinking water (86%) or sewage drainage (89%) [22]. Further, Maputo ranks as the province with the third highest HIV prevalence in the country (16.9%) [21].

The health centers were chosen because they are in densely populated and less urbanized areas, where housing conditions are precarious and basic socio-sanitary infrastructure is limited [23]. These factors increase the vulnerability of their residents to diarrheal diseases, making it a particularly important issue to study in these areas. Additionally, the choice of Maputo city was based on the limited availability of studies on *Salmonella*, *Shigella*, and *Campylobacter* in individuals with diarrhea in the city. Previous studies on enteric infections in Maputo city have primarily focused on *E*. *coli* [16, 17]. Furthermore, the only study that isolated a broad range of bacteria including *Salmonella*, *Shigella* and *Campylobacter* from stools of residents of the city was conducted specifically in densely populated, low-income, unplanned neighborhoods of Maputo and focused on children below four years with and without diarrhea [18]. In contrast, our study focuses specifically on patients of all ages visiting primary healthcare facilities with diarrhea, providing important new information on the prevalence of the three bacteria and risk factors for enteric infection in Maputo city.

### Population and eligibility criteria

The study’s participants included patients of all ages who presented to an outpatient consultation with diarrhea. We defined the cases as HIV-infected patients, while the controls were HIV-uninfected. We excluded patients who had undetermined HIV sero-status, those who did not provide stool samples or complete the questionnaire, those who reported taking antibiotics within seven days before recruitment, those who had undergone bowel surgery, and HIV-infected patients with clotted, hemolyzed or incomplete blood samples. (S1 Fig) presents the study flow chart. Diarrhea was defined as the passage of three or more loose or liquid stools per day, or more frequently than normal for the individual, in accordance with the WHO definition [24].

### Sample size and sampling technique

The sample size was determined based on the formula described below [25], using a 95% confidence interval and an expected prevalence of 25% for all pathogens.


$$n=\frac{\left(z^{2})P(1-P\right)}{d^{2}}$$

$$n=\frac{1.96^{2}\times 0.25\times (1-0.25)}{0.05^{2}}$$

n=288.12≈288

Where:

n = sample number

z = statistic for the confidence level

P = expected prevalence

d = allowed margin of error

To increase the precision of the study, a sample size of 300 was recruited. Thus, for each health center, a total of 150 patients divided into 75 HIV-infected patients and 75 HIV-uninfected patients were recruited. Patients who met the inclusion criteria were recruited until the sample size was reached.

### Data collection

The research team used a semi-structured questionnaire to collect socio-demographic characteristics, including age, sex, residence, nationality, place of birth, education level, employment/occupation, type of food consumption, source of drinking water, use of boiled water, use of latrine, cohabitation with pets (dogs and/or cats) and cohabitation with chickens and/or ducks. Clinical information related to HIV sero-status, as well as signs and symptoms presented by the patients, such as vomiting, fever (>38°C), weakness, dehydration, cough, abdominal pain and nausea were also collected. Additionally, data on the history of antibiotic intake in the previous 15 days were recorded. Each study participant’s weight and height were measured using a weighing scale and a stadiometer, respectively, and the Body Mass Index (BMI) was calculated according to WHO guidelines [26, 27].

We requested each patient to provide a stool sample in a sterile, disposable plastic container for stool specimens within 24h. The stool samples were placed in Cary-Blair transport medium, labeled with unique study identification numbers, and transported to the Microbiology Laboratory of the Faculty of Sciences at Eduardo Mondlane University (UEM) to be processed within 2 hours from collection.

Furthermore, for HIV-infected patients, we collected 4 ml of blood via venipuncture and placed it into a tube with Ethylenediaminetetraacetic acid (EDTA) [28]. The blood samples were transported at 4°C to the Parasitology Laboratory at the UEM Faculty of Medicine for further processing to measure the HIV-1 viral load.

### Laboratory analysis

#### Microbiological analysis and culture

To isolate *Salmonella* and *Shigella*, a loopful of each stool sample was cultured in Selenite F Broth at 37°C for 24h followed by subculturing onto *Salmonella-Shigella* agar (Condalab, Spain) at 37°C for 24h. On *Salmonella-Shigella* agar, *Salmonella* spp. colonies appeared colorless with a black center, while *Shigella* spp. colonies were colorless. Antisera from the *Salmonella* seroquick ID kit (SSI Diagnostica, Denmark) were used for further serotyping of *Salmonella typhimurium* and *enteritidis*. *Campylobacter* spp. were isolated for 48h at 42°C in microaerophilic conditions generated by Campygen sachets (Oxoid, UK) on Karmali agar medium enriched with selective Karmali supplement (Condalab, Spain). Catalase, hippurate hydrolysis, the H_2_S test, and gram stain were used to identify and differentiate *C*. *jejuni* and *C*. *coli* colonies grown on Karmali agar.

#### Determination of plasma HIV-1 RNA level

Upon arrival at the Parasitology Laboratory, the collected blood samples were centrifuged at 150 r.p.m. for 5 minutes to extract plasma, which was subsequently stored at -20°C. Purified RNA was obtained from a 0.5 mL plasma sample and the quantitative real-time PCR (qRT-PCR) amplification and detection were done in the m2000 platform using Abbott RealTime HIV-1 assay for viral load measurements according to the manufacturer’s instructions (https://www.molecular.abbott/int/en/products/infectious-disease/realtime-hiv-1-viral-load). The assay has an analytical sensitivity of 75 HIV-1 RNA copies/mL. Moreover, for a subset of 37 samples that had PCR run errors in the Abbott assay, the HIV-1 viral load measurement was performed using Roche COBAS® AmpliPrep/COBAS® TaqMan® HIV-1 Test, v2.0., at the Mavalane General Hospital. Purified RNA was obtained from 1 ml plasma aliquots and detection was performed using the COBAS® 6800 system according to the manufacturer’s instructions (https://diagnostics.roche.com/global/en/products/params/cobas-ampliprep-cobas-taqman-hiv-1-test-v2-0.html#productSpecs). The assay has an analytical sensitivity of 20 HIV-1 RNA copies/mL.

### Data analysis

Data were coded and entered into an Excel 2019 database before being analyzed with Epi Info™ version 7.2.5.0 from the Centers for Disease Control and Prevention (CDC). Before statistical analysis, all data were checked for eligibility, completeness, and consistency. The age categories were adapted from Mozambique census. Moreover, the severity of enteric infection symptoms was grouped in three categories, namely: the presence of 0–1; 2–3 and ≥ 4 symptoms (among the following: vomiting, fever, tiredness/weakness, dehydration, cough, abdominal pain and nausea). The HIV-1 viral load was categorized as >1,000 copies/ml (virological failure) and <1,000 copies/ml (low-level viraemia) according to the WHO categories [29].

The data were summarized using descriptive statistics. Bivariate logistic regression was used to examine the relationship between the selected risk factors (predictor variables) and bacterial infection (outcome variable). Variables with a p-value of <0.25 in the bivariate logistic regression were chosen for further analysis in a multivariate logistic regression model to control for potential confounding factors. The results were presented using the crude odds ratio (COR), adjusted odds ratio (AOR), and 95% level of confidence. We compared the prevalence of bacterial infections by age groups among patients using both the Chi-square test and Fisher’s exact test, as appropriate. A p-value of <0.05 was considered statistically significant.

### Ethical considerations

This study was approved by Mozambique’s National Bioethical Committee for Health (IRB00002657, reference No: 48/CNBS/21) and followed the principles of the Helsinki Declaration. Before any patient participated in the research, written informed consent was obtained. Participants over the age of 18 signed their consent. Written informed consent was obtained from the parent or legal guardian of each participant under 18 years of age. Besides parental consent, participants aged 12 to 17 years provided their assent. Illiterate participants added their fingerprint to the consent form along with an impartial witness who also signed and was present during the informed consent process. We kept the information and test results of all study participants strictly confidential. The results of bacterial cultures and viral load analysis were reported to the clinician responsible for patient care. Additionally, the filled PLOS’ questionnaire on inclusivity in global research (included in S1 File) was completed, outlining ethical, cultural, and scientific considerations specific to inclusivity in global research.

## Results

### Sociodemographic characteristics of study participants

As shown in Table 1, both the HIV-infected and uninfected groups were predominantly female (66.7% and 62.7%, respectively). The age distribution of patients differed between the two groups, with the highest proportion of HIV-infected patients falling within the 30–44-year age group (53.3%) and the highest proportion of HIV-uninfected patients falling within the 0–14-year age group (35.5%). Most patients in both groups lived in suburban or peri-urban areas (92% and 93.3%, respectively). Furthermore, the majority of patients in both groups reported consuming chicken, drinking tap water, and boiling their drinking water.

**Table 1:** Socio–demographic data of study participants.

Characteristics	Categories	Total n (%)	HIV infected n (%)	HIV uninfected n (%)	p-value
Sex	Female	194 (64.7)	100 (66.7)	94 (62.7)	0.468
	Male	106 (35.3)	50 (33.3)	56 (37.3)	
Age	0–14	55 (18.3)	2 (1.3)	53 (35.3)	**0.000** *
	15–29	56 (18.7)	14 (9.3)	42 (28.0)	
	30–44	101 (33.7)	80 (53.3)	21 (14.0)	
	45–59	54 (18.0)	42 (28.0)	12 (8.0)	
	≥60	34 (11.3)	12 (8.0)	22 (14.7)	
Residence	Urban/under urbanization	22 (7.3)	12 (8.0)	10 (6.7)	0.658
	Suburban/peri-urban	278 (92.7)	138 (92.0)	140 (93.3)	
Education levelª	Secondary or higher	57 (23.3)	28 (18.9)	29 (29.9)	**0.007** *
	Basic level	94 (38.4)	62 (41.9)	32 (33.0)	
	Basic level incomplete	82 (33.5)	55 (37.2)	27 (27.8)	
	Illiterate	12 (4.9)	3 (2.0)	9 (9.3)	
Occupationª	Employed	121 (49.4)	80 (54.1)	41 (42.3)	0.071
	Unemployed	124 (50.6)	68 (45.9)	56 (57.7)	
Consumption of chicken	No	95 (31.7)	53 (35.3)	42 (28.0)	0.172
	Yes	205 (68.3)	97 (64.7)	108 (72.0)	
Consumption of eggs	No	143 (47.7)	81 (54.0)	62 (41.3)	**0.028** *
	Yes	157 (52.3)	69 (46.0)	88 (58.7)	
Consumption of raw vegetables	No	164 (54.7)	89 (59.3)	75 (50.0)	0.104
	Yes	136 (45.3)	61 (40.7)	75 (50.0)	
Source of drinking water	Tap	254 (84.7)	134 (89.3)	120 (80.0)	0.074
	Public standpipe/well	25 (8.3)	8 (5.3)	17 (11.3)	
	Mineral and/or tap	21 (7.0)	8 (5.3)	13 (8.7)	
Boiling of drinking water	Yes	75 (25.0)	42 (28.0)	33 (22.0)	0.230
	No	225 (75.0)	108 (72.0)	117 (78.0)	
Use of latrine^a^^,^^b^	No	73 (29.8)	43 (29.1)	30 (30.9)	0.754
	Yes	172 (70.2)	105 (70.9)	67 (69.1)	
Cohabitation with pets (dogs and/or cats)	No	162 (54.0)	89 (59.3)	73 (48.7)	0.064
	Yes	138 (46.0)	61 (40.7)	77 (51.3)	
Cohabitation with chickens and/or ducks	No	215 (71.7)	107 (71.3)	108 (72.0)	0.898
	Yes	85 (28.3)	43 (28.7)	42 (28.0)	
Viral load range	<1,000	128 (86.5)	128 (86.5)	–	–
	>1,000	20 (13.5)	20 (13.5)	–	
Severity of symptoms	0–1	148 (49.3)	78 (52.0)	70 (46.7)	0.398
	2–3	117 (39.0)	58 (38.7)	59 (39.3)	
	≥ 4	35 (11.7)	14 (9.3)	21 (14.0)	
BMI	Normal	204 (68.0)	91 (60.7)	113 (75.3)	**0.024** *
	Underweight	27 (9.0)	16 (10.7)	11 (7.3)	
	Overweight	69 (23.0)	43 (28.7)	26 (17.3)	

ª Only participants aged above 15 years were included in the analysis for this parameter

^b^ instead of a toilet.

* p<0.05

It is noteworthy that patients aged above 15 years without HIV-1 infection were more likely to have had a secondary or higher education (29.9%) than those who were infected (18.9%) (p = 0.00). Recent egg consumption was reported by more HIV-uninfected patients (58.7%, p = 0.028). Furthermore, overweight patients were more likely to be HIV-infected (28.7%, p = 0.024).

In the study, HIV-1 RNA level data was available for a total of 148 patients. Among them, 115 (77.7%) had copy numbers equal to or below 75, while 13 patients had levels ranging between 76 and 1,000. The remaining 20 patients had an average HIV-1 RNA level of 327,218.45, with a maximum value of 3,801,893 copies/ml. For more detailed information, please refer to S1 Table, which presents the study database.

### Prevalence of enteric bacteria

Table 2 summarizes the prevalence and comparisons of the three bacteria in HIV-infected and -uninfected patients. Of the total 300 patients in our study, consisting of both HIV-infected and uninfected individuals, we observed that 129 patients (43.0%) had at least one bacterial infection. The prevalence of bacterial infections was slightly higher in HIV-infected patients (45.3%) compared to HIV-uninfected patients (40.7%), although this difference was not statistically significant. Only *Shigella* spp. was found to be associated with HIV (p = 0.038), with HIV- infected patients having a higher prevalence (19.3%) compared to HIV-uninfected patients (10.7%).

**Table 2:** Prevalence of *Salmonella* spp., *Shigella* spp. *and Campylobacter* spp. among study participants.

Parameter	Total n (%)	HIV infected n (%)	HIV uninfected n (%)	HIV infected vs HIV uninfected	
				OR (95 CI)	p-value
Any bacterial infection	129 (43.0)	68 (45.3)	61 (40.7)	1.2 (0.8–1.9)	0.414
*Salmonella* spp.	99 (33.0)	46 (30.7)	53 (35.3)	0.8 (0.5–1.3)	0.390
*Salmonella typhimurium*	5 (1.7)	3 (2.0)	2 (1.3)	1.5 (0.2–9.2)	0.654
*Salmonella enteritidis*	14 (4.7)	6 (4.0)	8 (5.3)	0.7 (0.3–2.2)	0.585
Other *Salmonella*	80 (26.7)	37 (24.7)	43 (28.7)	0.8 (0.5–1.4)	0.434
*Shigella* spp.	45 (15.0)	29 (19.3)	16 (10.7)	2.0 (1.0–3.9)	**0.038** ^*^
*Campylobacter* spp.	13 (4.3)	7 (4.7)	6 (4.0)	1.2 (0.4–3.6)	0.777
*Campylobacter coli*	12 (4.0)	7 (4.7)	5 (3.3)	1.4 (0.4–4.6)	0.558
*Campylobacter jejuni*	1 (0.3)	0 (0)	1 (0.7)	–	0.973
Mixed infections					
*Salmonella*/*Shigella*	19 (6.3)	9 (6.0)	10 (6.7)	0.9 (0.4–2.3)	0.813
*Salmonella/Shigella/Campylobacter*	1 (0.3)	1 (0.7)	0 (0)	–	0.973
*Salmonella/Campylobacter*	6 (2.0)	2 (1.3)	4 (2.7)	0.5 (0.1–2.7)	0.419
*Shigella/Campylobacter*	1 (0.3)	1 (0.7)	0 (0)	–	0.973

^a^ Other than *Salmonella typhimurium and Salmonella enteritidis*

*p<0.05

*Salmonella* spp. was the most prevalent bacterial species identified in the study population with a prevalence rate of 33.0%, whereas *Campylobacter* spp. was found to have the lowest prevalence rate at 4.3%. The majority of mixed infections (6.3%) were attributed to *Salmonella* and *Shigella*.

The S2 File presents our findings on the prevalence of bacterial infections among different age groups and HIV status. The prevalence of bacterial infections varied significantly across age groups, with the highest prevalence observed in patients aged below 5 years (56.3%) and those aged between 30–44 years (52.5%) (p = 0.04). No significant differences were found in HIV-infected and uninfected patients when analyzing age ranges.

While not statistically significant, we observed lower prevalence of *Salmonella*, *Shigella*, and *Campylobacte*r in patients aged above 60 years (23.5%, 5.9%, and 2.9%, respectively). Additionally, age range did not show significant differences in the prevalence of *Salmonella* and *Campylobacter* in both HIV infected and uninfected patients.

Our study found that the prevalence of *Shigella* spp. was highest in patients aged 30–44 years and those aged below 5 (23.8% and 21.9%, respectively) (p = 0.02). However, we did not find significant differences among HIV-infected and uninfected age ranges for this bacterium.

### Logistic regression of factors associated with a bacterial infection

Table 3 shows that based on bivariate logistic regression analysis, individuals in the 30–44 year age group (COR: 2.65, 95% CI: 1.15–6.10; p = 0.022), those with a basic education (COR: 2.39, 95% CI: 1.21–4.72); p = 0.012), those cohabiting with pets such as dogs and/or cats) (COR: 1.80, 95% CI: 1.13–2.86, p = 0.013), and those presenting with two or three symptoms of enteric infection (COR: 2.30, 95% CI: 1.40–3.78, p = 0.001) had a higher likelihood of having a bacterial pathogen detected in their stool.

**Table 3:** Bivariate analysis of the association between selected risk factors and enteric bacteria prevalence among patients.

Variables	Categories	Enteric infection			Univariate logistic analysis	
		N. tested	N. positive	% prevalence	COR (95% CI)	p-value
Sex	Female	194	89	45.9	1	–
	Male	106	40	37.7	0.71 (0.44–1.16)	0.174
Age	≥60	34	10	29.4	1	–
	45–59	54	20	37.0	1.41 (0.56–3.55)	0.464
	30–44	101	53	52.5	2.65 (1.15–6.10)	**0.022** *
	15–29	56	21	37.5	1.44 (0.58–3.59)	0.435
	0–14	55	25	45.5	2.00 (0.81–4.96)	0.135
Residence	Urban/Under urbanization	22	9	40.9	1	–
	Suburban/Peri-urban	278	120	43.2	1.10 (0.45–2.65)	0.838
Education level^a^	Secondary/ higher	57	20	35.1	1	–
	Basic complete	94	53	56.4	2.39 (1.21–4.72)	**0.012** *
	Basic incomplete	82	26	31.7	0.85 (0.42–1.76)	0.677
	Illiterate	12	5	41.7	1.32 (0.37–4.71)	0.667
Occupation^a^	Employed	121	53	43.8	1	–
	Unemployed	124	51	41.1	0.90 (0.54–1.49)	0.672
Consumption of chicken	No	95	44	46.3	1	–
	Yes	205	85	41.5	0.82 (0.50–1.34)	0.430
Consumption of eggs	No	143	58	40.6	1	–
	Yes	157	71	45.2	1.21 (0.77–1.91)	0.415
Consumption of raw vegetables	No	164	72	43.9	1	–
	Yes	136	57	41.9	0.92 (0.58–1.46)	0.729
Source of drinking water	Tap	254	113	44.5	1	–
	Pub. standpipe/well	25	10	40.0	0.83 (0.36–1.92)	0.667
	Mineral and/or tap	21	6	28.6	0.50 (0.19–1.33)	0.164
Boiling of drinking water	Yes	75	35	46.7	1	–
	No	225	94	41.8	0.82 (0.48–1.39)	0.459
Use of latrine^a^^,^^b^	No	73	30	41.1	1	–
	Yes	172	74	43.0	1.08 (0.62–1.89)	0.780
Cohabitation with pets (dogs and/or cats)	No	162	59	36.4	1	–
	Yes	138	70	50.7	1.80 (1.13–2.86)	**0.013** *
Cohabitation with chickens and/or ducks	No	215	85	39.5	1	–
	Yes	85	44	51.8	1.64 (0.99–2.72)	0.055
HIV	No	150	61	40.7	1	–
	Yes	150	68	45.3	1.21 (0.77–1.91)	0.415
Viral load range	<1,000	128	57	44.5	1	–
	>1,000	20	10	50.0	1.24 (0.49–3.20)	0.647
Severity of symptoms	0–1	148	51	34.5	1	–
	2–3	117	64	54.7	2.30 (1.40–3.78)	**0.001** *
	≥ 4	35	14	40.0	1.27 (0.60–2.70)	0.539
BMI	Normal	204	92	45.1	1	–
	Underweight	27	9	33.3	0.61 (0.26–1.42)	0.250
	Overweight	69	28	40.6	0.83 (0.48–1.45)	0.514

* p<0.05

ª Only participants aged above 15 years were included in the analysis for this parameter.

^b^ instead of a toilet.

According to the multivariate analysis, the severity of symptoms and education level were risk factors for bacterial infection (p<0.05). Patients presenting 2–3 symptoms were found to be twice as likely to have a bacterial infection compared to those with 0–1 symptoms (AOR: 2.27, 95% CI: 1.23–4.15; p = 0.008). Moreover, compared with patients with secondary or higher education, patients with basic education were twice as likely to develop a bacterial infection (AOR: 2.26, 95% CI: 1.08–4.72, p = 0.030) (see Table 4).

**Table 4:** Results of multivariate logistic regression analysis of potential risk factors and their association with prevalence of enteric bacteria among patients with diarrhea.

Variables	Categories	Enteric infection		COR (95%CI)	p-value	AOR (95%CI)	p-value
		Negative	Positive				
Severity of symptoms	0–1	97 (65.5)	51 (34.5)	1	–	1	–
	2–3	53 (45.3)	64 (54.7)	2.30 (1.40–3.78)	**0.001** *	2.27 (1.23–4.15)	**0.008** **
	≥ 4	21 (60.0)	14 (40.0)	1.27 (0.60–2.70)	0.539	1.52 (0.62–3.72)	0.362
Education level^a^	Secondary/ higher	37 (64.9)	20 (35.1)	1	–	1	–
	Basic complete	41 (43.6)	53 (56.4)	2.39 (1.21–4.72)	**0.012** *	2.26 (1.08–4.72)	**0.030** **
	Basic incomplete	56 (68.3)	26 (31.7)	0.85 (0.42–1.76)	0.677	0.74 (0.33–1.69)	0.487
	Illiterate	7 (58.3)	5 (41.7)	1.32 (0.37–4.71)	0.067	1.01 (0.24–4.29)	0.989
Cohabitation with pets (dogs and/or cats)	No	103 (63.6)	59 (36.4)	1	–	1	–
	Yes	68 (49.3)	70 (50.7)	1.80 (1.13–2.86)	**0.013** *	1.56 (0.88–2.76)	0.124
Age	≥60	24 (70.6)	10 (29.4)	1	–	1	–
	45–59	34 (63.0)	20 (37.0)	1.41 (0.56–3.55)	0.464	1.08 (0.39–2.97)	0.878
	30–44	48 (47.5)	53 (52.5)	2.65 (1.15–6.10)	0.022	2.05 (0.79–5.29)	0.139
	15–29	35 (62.5)	21 (37.5)	1.44 (0.58–3.59)	0.435	0.86 (0.28–2.60)	0.781
	0–14	30 (54.5)	25 (45.5)	1.99 (0.81–4.96)	0.135	1.72 (0.90–3.30)	0.101
Cohabitation with chickens and/or ducks	No	130 (60.5)	85 (39.5)	1	–	1	–
	Yes	41 (48.2)	44 (51.8)	1.64 (0.99–2.72)	0.055	1.72 (0.90–3.30)	0.100
Source of drinking water	Tap	141 (55.5)	113 (44.5)	1	–	1	–
	Pub. standpipe/well	15 (60.0)	10 (40.0)	0.83 (0.36–1.92)	0.667	1.30 (0.47–3.62)	0.616
	Mineral and/or tap	15 (71.4)	6 (28.6)	0.50 (0.19–1.33)	0.164	1.05 (0.31–3.54)	0.940
Sex	Female	105 (54.1)	89 (45.9)	1	–	1	–
	Male	66 (62.3)	40 (37.7)	0.71 (0.44–1.16)	0.174	0.70 (0.37–1.33)	0.276
BMI	Normal	112 (54.9)	92 (45.1)	1	–	1	–
	Underweight	18 (66.7)	9 (33.3)	0.61 (0.26–1.42)	0.250	0.71 (0.26–1.89)	0.489
	Overweight	41 (59.4)	28 (40.6)	0.83 (0.48–1.45)	0.514	0.80 (0.41–1.53)	0.494

ª Only participants aged above 15 years were included in the analysis for this parameter.

* p<0.05 in bivariate analysis

** p<0.05 in multivariate analysis

S3 File provides the detailed results of bivariate and multivariate logistic regression analyses for *Salmonella* spp., *Shigella* spp. and *Campylobacter* spp. The bivariate analysis revealed that cohabitation with pets (dogs and/or cats), and presenting 2–3 symptoms were associated with *Salmonella* spp. infection (p<0.05). These results were consistent with those of the multivariate analysis, which demonstrated that patients with 2–3 symptoms were three times more likely to develop *Salmonella* spp. infection than patients with 0–1 symptom (AOR: 3.17; 95% CI: 1.68–5.98; p = 0.000). Additionally, patients who had pets (dogs and/or cats) at home were twice as likely to have a *Salmonella* spp. infection than those who did not (AOR: 2.02, 95% CI: 1.13–3.60, p = 0.018).

Regarding *Shigella* spp. infection, age and HIV infection were significant predictors in the bivariate analysis (p<0.05). However, in the multivariate model, none of the risk factors were found to be independently associated with *Shigella* spp. infection. Furthermore, neither the bivariate nor the multivariate models showed any predictor variables to be associated with *Campylobacter* spp. infection.

## Discussion

The study’s findings are relevant to the local context of Maputo, where the study was conducted, and highlight the importance of addressing the issue of bacterial infections among patients with diarrhea, particularly in peri-urban and suburban areas where basic infrastructure and access to clean water and sanitation may be lacking. In point of fact, only 48.7% of the inhabitants in Mozambique have access to safe drinking water and 54.2% do not have latrines in their homes [22, 30], which increases the risk of diarrheal diseases caused by enteric pathogens.

The higher prevalence of bacterial infections among children under five and patients aged 30–44 years is a cause for concern, especially as these age groups are likely to be more vulnerable to the health impacts of diarrheal diseases. There are several potential causes for these findings, which may include inadequate access to clean water and sanitation, poor hygiene practices, and inadequate food safety practices. In peri-urban and suburban areas where basic infrastructure may be lacking, access to clean water and sanitation may be limited, increasing the risk of bacterial infections. Poor hygiene practices such as not washing hands properly after using the toilet, handling food or changing diapers may also contribute to the spread of bacteria [31].

Moreover, in the study population, many may rely on informal street vendors or small-scale food vendors for their food, which may not have adequate food safety standards in place [32–34]. This may increase the risk of exposure to contaminated food, which can result in bacterial infections.

Our findings suggests that interventions such as health education campaings should be targeted towards the most affected age groups, which may include increased access to clean water and sanitation, improved food safety and hygiene practices, and targeted health education campaigns.

While not statistically significant, the prevalence of bacterial infections was higher in females compared to males. This observation could be attributed to a variety of factors. Women may have different hygiene practices or may be more likely to engage in activities that increase their risk of enteric infection. Furthermore, in Mozambique, it is customary for women to take on responsibilities such as cleaning cloth diapers used by children, managing children’s fecal matter, and picking up feces left by children who defecate in the household yard, leading to a higher likelihood of bacterial infections [35]. Additionally, women and girls may face greater socioeconomic challenges, such as poverty and limited education, which can increase their vulnerability to bacterial infections.

Comparing our results with previous studies conducted in Mozambique, it was found that the prevalence of *Salmonella* spp. in patients under 5 years this study (40.6%) was much higher than the 2.5% reported in 2007 among children of the same age with diarrhea in Manhiça district [8]. Additionally, other studies reported non-typhoidal *Salmonella* with a low prevalence ranging from 0 to 1% [11, 13, 15]. These differences could be due to the fact that the studies used different definitions of diarrhea and employed different diagnostic methods and designs. Moreover, our study was conducted in peri-urban and suburban areas of Maputo city, while those previously reported were all conducted in the Manhiça district of Maputo province [8–15]. We found that *Salmonella* was the most prevalent bacteria in this study when compared to *Shigella* and *Campylobacter*. Therefore, these results suggest that *Salmonella* is an important pathogen found in patients of all ages with diarrhea in the peri-urban and suburban areas of Maputo.

The prevalence of *Shigella* spp. in children under the age of five (21.9%) observed in this study was higher compared to previous studies from Mozambique in patients with diarrhea. In those studies, the prevalence ranged from 1 to 8% in children below the age of five [9, 11, 13, 15]. On the other hand, the low prevalence of *Campylobacter* spp. (4.3%) observed in this study is consistent with previous studies from Mozambique [8, 11, 13, 15], as well as with a pooled prevalence of 10.2% reported in patients with diarrhea from sub-Saharan Africa [36].

When comparing our results with those from other studies from sub-Saharan Africa, in a recent study conducted in Nandi County, Kenya, a similar prevalence of *Shigella* spp. was found (20.1%), although *C*. *jejuni* had a higher prevalence (12.9%) in children under the age of five with diarrhea [37]. Contrary to this finding, *Campylobacter* spp., *Salmonella* spp., and *Shigella* spp. had a prevalence of less than 4% in a similar study conducted in Nairobi [38].

In Ethiopia, a study of HIV-infected patients with diarrhea found a similar prevalence of *Campylobacter* spp. (4.4%), although *Salmonella* spp. and *Shigella* spp. had a much lower prevalence (2.1% and 1.1%, respectively) [39]. The prevalence of *Campylobacter* and *Shigella* spp. were similar in a study conducted in our neighboring country, South Africa (5% and 17%, respectively) in children under the age of 12 with diarrhea, though *Salmonella enterica* had a much lower prevalence (6%) [40]. These findings show that, occasionally, bacterial prevalence in patients with diarrhea is comparable in other sub-Saharan African countries, despite some fluctuations caused by a variety of factors including the variation in the predominance of pathogens depending on the location, the different sources of contamination to which the population is exposed and different hygienic-sanitary conditions [41]. Furthermore, the individual susceptibility to bacterial pathogens [42] and the immunological status of each individual [43] may also influence the colonization by enteric bacteria.

Now analyzing the association between bacterial pathogens and HIV infection, the bivariate logistic regression model revealed that *Shigella* spp. were the only bacteria significantly associated with HIV infection (p<0.05). However, in the multivariate analysis, this association was not significant. In keeping with this, a study from four years ago in Mozambique found that HIV-infected patients with diarrhea were more likely to present a high prevalence of *Shigella* spp. when compared with HIV-uninfected patients [12].

Few studies have compared the prevalence of a wide range of bacteria associated with diarrhea in HIV-infected and -uninfected patients. Among these, in an Ethiopian study, *Salmonella* spp. had a higher prevalence in HIV-infected patients, while the other two bacteria had the opposite result [44]. In India, *Campylobacter* spp. and *Shigella* spp. were more prevalent in HIV-infected patients [45]. On the other hand, none of the three bacteria were associated with HIV in an older study conducted in Kenya [46], and neither were *Salmonella* spp. nor *Shigella* spp. associated with HIV in another study conducted in Ethiopia [47]. Conversely, different results were found in studies that focused on a single pathogen, such as a lack of association between *Campylobacter* spp. infection and HIV in Tanzania [48], while this association was reported in another study conducted in South Africa [49].

Although some studies have reported an association between HIV infection and the presence of enteric bacteria, it is important to consider the potential factors that may have influenced the results of the current study. One such factor could be the therapeutic success in HIV-infected patients, which may have contributed to patients improved immune function. This is evidenced by the fact that 128 out of 148 patients with viral loads analyzed had HIV-1 RNA plasma levels below 1,000 copies/ml, indicating a low-level viraemia. Nonetheless, further investigations are needed to better understand the relationship between HIV and enteric pathogens in the context of different populations, treatment regimens, and other contributing factors.

Regarding the etiology of diarrhea in this study, it is important to consider that other bacterial, viral, and parasitic pathogens may have also played a causal role. For example, rotavirus is known to be highly prevalent in children under the age of five with diarrhea in Mozambique [4]. Additionally, *Giardia*, *Cryptosporidium*, and other enteric pathogens have been identified as etiologic agents of diarrhea in this country [6, 11].

In terms of factors associated with an enteric infection, our findings revealed that patients presenting with 2–3 symptoms of enteric infection were more likely to have a higher prevalence of bacterial infections, which is consistent with a previous study from Ethiopia [50]. Enteric pathogens commonly cause symptoms in addition to diarrhea, such as vomiting, fever, and abdominal pain [15, 51]. Another factor associated with enteric infections was education, likely due to the lower level of knowledge about disease prevention among those with lower education levels. As a matter of fact, in Mozambique, the adult literacy rate is 60.7% [52]. While awareness campaigns are already in place, such as that of the Educational Platform for Health Information (Plataforma Educativa de Informação Sobre a Saúde, PENSA) (https://www.pensa.org.mz/), managed by the Ministry of Health, which sends short messages containing health-related information to customers of all mobile operators in the country, more efforts are needed to prevent enteric infections that result in diarrhea. For instance, campaigns could emphasize the importance of maintaining proper hygiene, drinking clean water, and washing hands frequently.

In our study, *Salmonella* emerged as the only bacterium with significant independently associated factors, as evidenced by multiple logistic regression, both in terms of symptomatology and the cohabitation with dogs and/or cats, which is consistent with previous findings [50]. *Salmonella* spp. are common in both humans and animals in Mozambique. Dogs and cats can carry and shed *Salmonella*, contributing to cross-contamination [53]. For this reason, it is crucial to promote hygiene and sanitation practices in households to prevent human exposure to bacteria eliminated through animal feces.

Although previous studies have identified several risk factors for enteric infection caused by *Salmonella* spp. and *Campylobacter* spp., such as cohabitation with chickens and/or ducks, food consumption, hygiene and sanitation [36, 54–58], this was not observed in the current study. This could be due to the particular conditions of the study population’s living environments and their contact with animals raised at home. In addition, food preparation and storage practices may also have contributed to the lack of statistical associations with enteric infections.

Outlining the strengths of this study, overall, it measured the prevalence and risk factors for three important enteric bacteria in HIV-infected and -uninfected patients with diarrhea in peri-urban and suburban health centers. The only comparable study in Mozambique was performed in Manhiça from 2010 to 2012, which found that HIV infection increased the risk of having moderate-to-severe diarrhea caused by *Shigella* in children below five years (p<0.05) [12]. In that study, *Campylobacter* and *Salmonella* were not analyzed. Thus, this study provides information related to the impact of HIV on the prevalence of infections caused by three important enteric bacteria. In addition, this study provides evidence of the impact of viral load on the prevalence of enteric bacterial infections in Mozambique. Although our study may not cover all aspects of clinical management of diarrhea, our observation that *Salmonella* was identified more frequently than *Shigella* and *Campylobacter* highlights its significant role as a cause of diarrhea in our local population, which is consistent with global data [59, 60]. Additionally, our study findings revealed that bacterial infections had an influence on the presentation of additional symptoms in patients, and that education level has an influence on enteric infection. These findings can support the establishment of diarrhea prevention protocols in our local context.

It is important to acknowledge that this study has some limitations. First, regarding HIV, it was not possible to record information about patients’ antiretroviral therapeutic regimens, which could have been a variable to be analyzed. Additionally, information about the prescribed treatment and the outcome of diarrheal disease was not available. Also, because the study was conducted during the fourth wave of COVID-19 in Mozambique, no information on the diagnosis of COVID-19 was obtained, which could have been an important predictor to examine in both bivariate and multivariate logistic regression models.

## Conclusions

In this study on the prevalence of *Salmonella*, *Shigella* and *Campylobacter* in association with HIV in Mozambique, we found that nearly half (43.0%) of the patients with diarrhea had at least one bacterial infection. *Salmonella* spp. had the highest prevalence (33.0%), followed by *Shigella* spp. (15.0%) and *Campylobacter* spp. (4.3%). Our study highlights the important role of *Salmonella* as a cause of diarrhea in our local population, which is consistent with global data. We also found that presentation with 2–3 symptoms, and education level associated with bacterial infection. Only *Shigella* spp. was associated with HIV in the bivariate logistic regression model (p<0.05), although not in the multivariate analysis. No association was detected between HIV viral load and the prevalence of bacteria in HIV-infected patients. Based on our results, we suggest that it may not be necessary to develop specific protocols for the clinical management of patients with co-occurring diarrhea and HIV, or according to viral load. However, further studies including other enteric pathogens will be important to provide a broader understanding of the prevalence of enteric infections in this population. To comprehend how HIV infection and immunosuppression status affect the prevalence of enteric bacteria, more in-depth research should be done and whenever possible, involving antiretroviral therapy.

## Supporting information

S1 Fig Study flow diagram. (TIF)

S1 Table Study database. (XLSX)

S1 File PLOS’ questionnaire on inclusivity in global research. (DOCX)

S2 File Prevalence of *Salmonella* spp., *Shigella* spp., and *Campylobacter* spp. by age group and HIV status. (DOCX)

S3 File Bivariate and multivariate logistic regression of association between risk factors and *Salmonell*a spp., *Shigella* spp. and *Campylobacter* spp. prevalence. (DOCX)
