# Supplementary figures and images for: Microbiological assessment reveals that Salmonella, Shigella and Campylobacter infections are widespread in HIV infected and uninfected patients with diarrhea in Mozambique
Source: PLOS Glob Public Health. 2023 May 22;3(5):e0001877. doi: 10.1371/journal.pgph.0001877 (PMC10202286; doi:10.1371/journal.pgph.0001877)

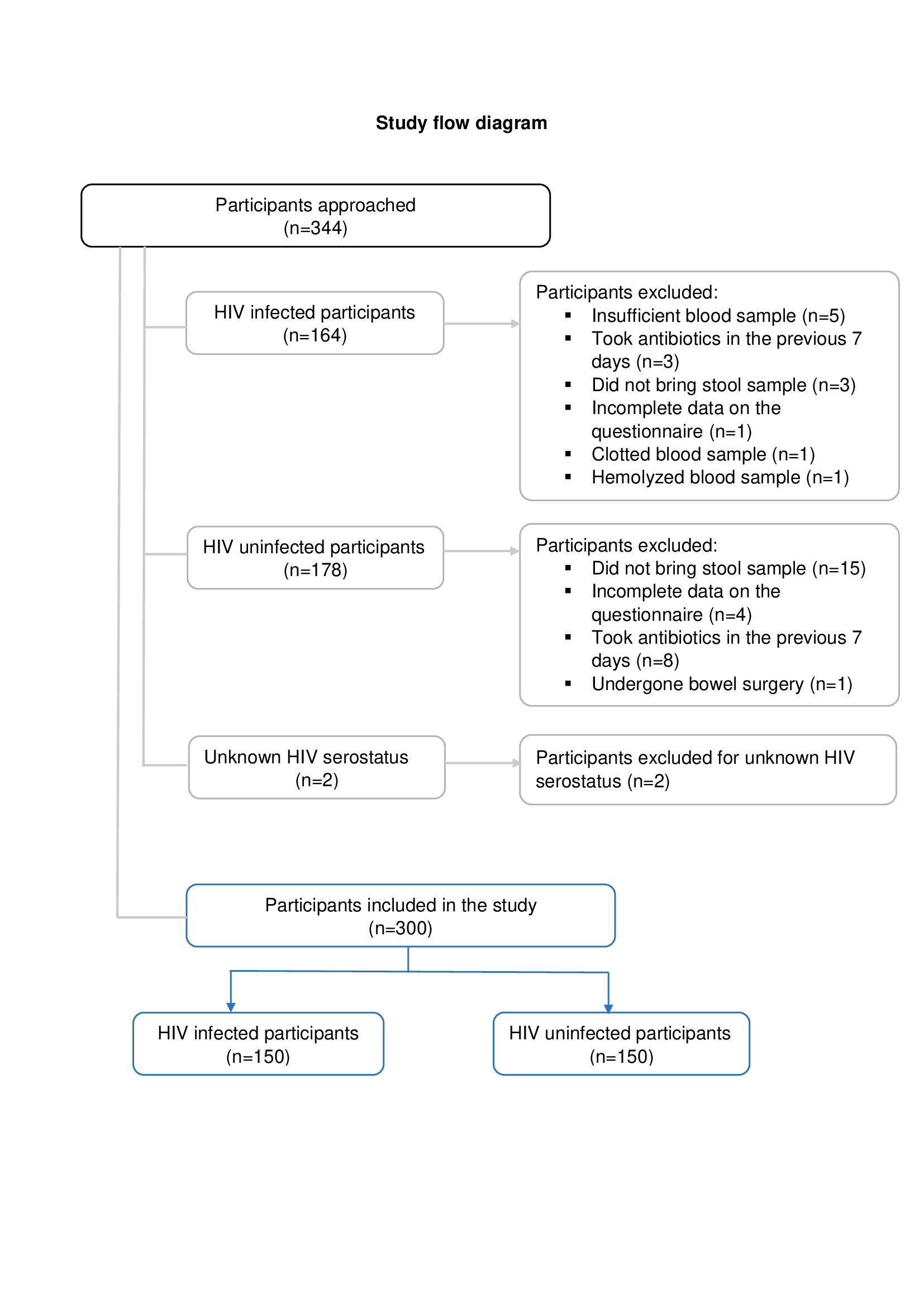

Supplement: S1 Fig — (TIF) [file pgph.0001877.s001.tif]
