# Supplementary material for: Microbiological assessment reveals that Salmonella, Shigella and Campylobacter infections are widespread in HIV infected and uninfected patients with diarrhea in Mozambique
Source: PLOS Glob Public Health. 2023 May 22;3(5):e0001877. doi: 10.1371/journal.pgph.0001877 (PMC10202286; doi:10.1371/journal.pgph.0001877)
Supplement: S2 File — (DOCX) [file pgph.0001877.s004.docx]

| **Microbiological assessment reveals that *Salmonella*, *Shigella* and *Campylobacter* infections are widespread in HIV infected and uninfected patients with diarrhea in Mozambique** |
| --- |

**Prevalence of *Salmonella* spp., *Shigella* spp., and *Campylobacter* spp. by age group and HIV status**

**Table 1.** Comparison of the prevalence of bacterial infections by age group among all patients.

| **Parameter** | **Age groups** | | | | | | **Chi-square** | **P-value** | **Fisher’s Exact** |
| --- | --- | --- | --- | --- | --- | --- | --- | --- | --- |
|  | **0–4** | **5–14** | **15–29** | **30–44** | **45–59** | **≥60** |  |  |  |
|  | (n=32) | (n=23) | (n=56) | (n=101) | (n=54) | (n=34) |  |  |  |
| Any bacteria | 18 (56.3%) | 7 (30.4%) | 21 (37.5%) | 53 (52.5%) | 20 (37.0%) | 10 (29.4%) | 11.509 | **0.042*** | **0.033*** |
| *Salmonella* spp. | 13 (40.6%) | 7 (30.4%) | 18 (32.1%) | 39 (38.6%) | 14 (25.9%) | 8 (23.5%) | 4.970 | 0.420 | - |
| *Salmonella typhimurium* | 1 (3.1%) | 1 (4.4%) | 0 (0%) | 1 (1.0%) | 2 (3.7%) | 0 (0%) | 4.599 | 0.467 | 0.296 |
| *Salmonella enteritidis* | 4 (12.5%) | 2 (8.7%) | 2 (3.6%) | 4 (4.0%) | 2 (3.7%) | 0 (0%) | 7.294 | 0.200 | 0.205 |
| Other *Salmonella* | 8 (25.0%) | 4 (17.4%) | 16 (28.6%) | 34 (33.7%) | 10 (18.5%) | 8 (23.5%) | 5.694 | 0.337 | - |
| *Shigella* spp. | 7 (21.9%) | 2 (8.7%) | 4 (7.1%) | 24 (23.8%) | 6 (11.1%) | 2 (5.9%) | 13.554 | **0.019*** | **0.022*** |
| *Campylobacter* spp. | 2 (6.3%) | 1 (4.4%) | 3 (5.4%) | 4 (3.9%) | 2 (3.7%) | 1 (2.9%) | 0.670 | 0.985 | 0.971 |
| *Campylobacter coli* | 2 (6.3%) | 1 (4.4%) | 2 (3.6%) | 4 (4.0%) | 2 (3.7%) | 1 (2.9%) | 0.568 | 0.989 | 0.982 |
| *Campylobacter jejuni* | 0 (0.0%) | 0 (0.0%) | 1 (1.8%) | 0 (0.0%) | 0 (0.0%) | 0 (0.0%) | 4.372 | 0.497 | 0.663 |

* p<0.05

**Table 2.** Comparison of the prevalence of bacterial infections by age group among HIV-infected patients.

| **Parameter** | **Age groups** | | | | | | **Chi-square** | **P-value** | **Fisher’s Exact** |
| --- | --- | --- | --- | --- | --- | --- | --- | --- | --- |
|  | **0–4** | **5–14** | **15–29** | **30–44** | **45–59** | **≥60** |  |  |  |
|  | (n=2) | (n=0) | (n=14) | (n=80) | (n=42) | (n=12) |  |  |  |
| Any bacteria | 1 (50%) | 0 (0%) | 5 (35.7%) | 42 (52.5%) | 17 (40.5%) | 3 (25.0%) | N/A | N/A | 0.299 |
| *Salmonella* spp. | 1 (50.0%) | 0 (0%) | 3 (21.4%) | 29 (36.3%) | 11 (26.2%) | 2 (16.7%) | N/A | N/A | 0.451 |
| *Salmonella typhimurium* | 0 (0%) | 0 (0%) | 0 (0%) | 1 (1.3%) | 2 (4.8%) | 0 (0%) | N/A | N/A | 0.610 |
| *Salmonella enteritidis* | 0 (0%) | 0 (0%) | 1 (7.1%) | 4 (5.0%) | 1 (2.4%) | 0 (0%) | N/A | N/A | 0.699 |
| Other *Salmonella* | 1 (50.0%) | 0 (0%) | 2 (14.3%) | 24 (30.0%) | 8 (19.1%) | 2 (16.7%) | N/A | N/A | 0.398 |
| *Shigella* spp. | 1 (50.0%) | 0 (0.0%) | 2 (14.3%) | 20 (25.0%) | 5 (11.9%) | 1 (8.3%) | N/A | N/A | 0.226 |
| *Campylobacter* spp. | 0 (0.0%) | 0 (0.0%) | 1 (7.1%) | 4 (5.0%) | 2 (4.8%) | 0 (0.0%) | N/A | N/A | 0.930 |
| *Campylobacter coli* | 0 (0.0%) | 0 (0.0%) | 1 (7.1%) | 4 (5.0%) | 2 (4.8%) | 0 (0.0%) | N/A | N/A | 0.930 |
| *Campylobacter jejuni* | 0 (0.0%) | 0 (0.0%) | 0 (0.0%) | 0 (0.0%) | 0 (0.0%) | 0 (0.0%) | N/A | N/A | 1.000 |

**Table 3.** Comparison of the prevalence of bacterial infections by age group among HIV-uninfected patients.

| **Parameter** | **Age groups** | | | | | | **Chi-square** | **P-value** | **Fisher’s Exact** |
| --- | --- | --- | --- | --- | --- | --- | --- | --- | --- |
|  | **0–4** | **5–14** | **15–29** | **30–44** | **45–59** | **≥60** |  |  |  |
|  | (n=30) | (n=23) | (n=42) | (n=21) | (n=12) | (n=22) |  |  |  |
| Any bacteria | 17 (56.7%) | 7 (30.4%) | 16 (38.1%) | 11 (52.4%) | 3 (25.0%) | 7 (31.8%) | 7.425 | 0.191 | 0.207 |
| *Salmonella* spp. | 12 (40%) | 7 (30.4%) | 15 (35.7%) | 10 (47.6%) | 3 (25.0%) | 6 (27.3%) | 3.104 | 0.684 | 0.711 |
| *Salmonella typhimurium* | 1 (3.3%) | 1 (4.4%) | 0 (0%) | 0 (0%) | 0 (0%) | 0 (0%) | 3.812 | 0.577 | 0.562 |
| *Salmonella enteritidis* | 4 (13.3%) | 2 (8.7%) | 1 (2.4%) | 0 (0%) | 1 (8.3%) | 0 (0%) | 7.679 | 0.175 | 0.149 |
| Other *Salmonella* | 7 (23.3%) | 4 (17.4%) | 14 (33.3%) | 10 (47.6%) | 2 (16.7%) | 6 (27.3%) | 6.849 | 0.232 | 0.269 |
| *Shigella* spp. | 6 (20.0%) | 2 (8.7%) | 2 (4.8%) | 4 (19.1%) | 1 (8.3%) | 1 (4.6%) | 6.855 | 0.232 | 0.238 |
| *Campylobacter* spp. | 2 (6.7%) | 1 (4.4%) | 2 (4.8%) | 0 (0.0%) | 0 (0.0%) | 1 (4.6%) | 2.018 | 0.847 | 0.967 |
| *Campylobacter coli* | 2 (6.7%) | 1 (4.4%) | 1 (2.4%) | 0 (0.0%) | 0 (0.0%) | 1 (4.6%) | 2.464 | 0.782 | 0.914 |
| *Campylobacter jejuni* | 0 (0.0%) | 0 (0.0%) | 1 (2.4%) | 0 (0.0%) | 0 (0.0%) | 0 (0.0%) | 2.589 | 0.763 | 1.000 |
