# Supplementary material for: Microbiological assessment reveals that Salmonella, Shigella and Campylobacter infections are widespread in HIV infected and uninfected patients with diarrhea in Mozambique
Source: PLOS Glob Public Health. 2023 May 22;3(5):e0001877. doi: 10.1371/journal.pgph.0001877 (PMC10202286; doi:10.1371/journal.pgph.0001877)
Supplement: S3 File — (DOCX) [file pgph.0001877.s005.docx]

| **Microbiological assessment reveals that *Salmonella*, *Shigella* and *Campylobacter* infections are widespread in HIV infected and uninfected patients with diarrhea in Mozambique** |
| --- |

**Bivariate and multivariate logistic regression of association between risk factors and *Salmonella* spp., *Shigella* spp. and *Campylobacter* spp. prevalence.**

**Table 1.** Bivariate analysis of association between selected risk factors and *Salmonella* spp. prevalence among study participants.

| **Variables** | **Categories** | ***Salmonella* spp.** | | | **Univariate logistic analysis** | |
| --- | --- | --- | --- | --- | --- | --- |
|  |  | **N. tested** | **N.**  **positive** | **%**  **prevalence** | **COR (95% CI)** | **p value** |
| Sex | Female | 194 | 68 | 35.1 | 1 | – |
|  | Male | 106 | 31 | 29.2 | 0.76 (0.46–1.28) | 0.307 |
| Age | ≥60 | 34 | 8 | 23.5 | 1 | – |
|  | 45–59 | 54 | 14 | 25.9 | 1.13 (0.42–3.09) | 0.801 |
|  | 30–44 | 101 | 39 | 38.6 | 2.04 (0.84–4.97) | 0.115 |
|  | 15–29 | 56 | 18 | 32.1 | 1.54 (0.58–4.06) | 0.384 |
|  | 0–14 | 55 | 20 | 36.4 | 1.86 (0.71–4.87) | 0.209 |
| Residence | Urban/Under urbanization | 22 | 7 | 31.8 | 1 | – |
|  | Suburban/Peri-urban | 278 | 92 | 33.1 | 1.06 (0.42–2.69) | 0.903 |
| Education level^a^ | Secondary/ higher | 57 | 16 | 28.1 | 1 | – |
|  | Basic complete | 94 | 41 | 43.6 | 1.98 (0.98–4.02) | 0.058 |
|  | Basic incomplete | 82 | 19 | 23.2 | 0.77 (0.36–1.67) | 0.514 |
|  | Illiterate | 12 | 3 | 25.0 | 0.85 (0.20–3.56) | 0.829 |
| Occupation^a^ | Employed | 121 | 41 | 33.9 | 1 | – |
|  | Unemployed | 124 | 38 | 30.7 | 0.86 (0.50–1.47) | 0.588 |
| Consumption of chicken | No | 95 | 34 | 35.8 | 1 | – |
|  | Yes | 205 | 65 | 31.7 | 0.83 (0.50–1.39) | 0.485 |
| Consumption of eggs | No | 143 | 46 | 32.2 | 1 | – |
|  | Yes | 157 | 53 | 33.8 | 1.07 (0.66–1.74) | 0.770 |
| Consumption of raw vegetables | No | 164 | 58 | 35.4 | 1 | – |
|  | Yes | 136 | 41 | 30.2 | 0.79 (0.48–1.28) | 0.339 |
| Source of drinking water | Tap | 254 | 87 | 34.3 | 1 | – |
|  | Public standpipe/well | 25 | 7 | 28.0 | 0.75 (0.30–1.86) | 0.529 |
|  | Mineral and/or tap | 21 | 5 | 23.8 | 0.60 (0.21–1.69) | 0.334 |
| Boiling of drinking water | Yes | 75 | 23 | 30.7 | 1 | – |
|  | No | 225 | 76 | 33.8 | 1.15 (0.66–2.03) | 0.620 |
| Use of latrine^a,b^ | No | 73 | 22 | 30.1 | 1 | – |
|  | Yes | 172 | 57 | 33.1 | 1.15 (0.64–2.08) | 0.646 |
| Cohabitation with pets (dogs and/or cats) | No | 162 | 43 | 26.5 | 1 | – |
|  | Yes | 138 | 56 | 40.6 | 1.89 (1.16–3.08) | **0.010*** |
| Cohabitation with chickens and/or ducks | No | 215 | 67 | 31.2 | 1 | – |
|  | Yes | 85 | 32 | 37.7 | 1.33 (0.79–2.26) | 0.282 |
| HIV | No | 150 | 53 | 35.3 | 1 | – |
|  | Yes | 150 | 46 | 30.7 | 0.81 (0.50–1.31) | 0.390 |
| Viral load range | <1,000 | 128 | 40 | 31.3 | 1 | – |
|  | >1,000 | 20 | 5 | 25.0 | 0.73 (0.25–2.16) | 0.573 |
| Severity of symptoms | 0–1 | 148 | 31 | 21.0 | 1 | – |
|  | 2–3 | 117 | 56 | 47.9 | 3.46 (2.03–5.93) | **0.000*** |
|  | ≥ 4 | 35 | 12 | 34.3 | 1.97 (0.88–4.39) | 0.098 |
| BMI | Normal | 204 | 71 | 34.8 | 1 | – |
|  | Underweight | 27 | 7 | 25.9 | 0.66 (0.26–1.63) | 0.362 |
|  | Overweight | 69 | 21 | 30.4 | 0.82 (0.46–1.48) | 0.507 |

^a^ Only patients above the age of 15 were included in the analysis for this parameter

^b^ instead of toilet

* p<0.05 in bivariate analysis

**Table 2.** Multivariate logistic regression analysis of potential risk factors and their association with prevalence of *Salmonella* spp. among study participants.

| **Variables** | **Categories** | ***Salmonella* spp.** | | **COR (95%CI)** | **p–value** | **AOR (95%CI)** | **p–value** |
| --- | --- | --- | --- | --- | --- | --- | --- |
|  |  | **Negative (%)** | **Positive (%)** |  |  |  |  |
| Severity of symptoms | 0–1 | 117 (79.0) | 31 (21.0) | 1 | – | 1 | – |
|  | 2–3 | 61 (52.1) | 56 (47.9) | 3.46 (2.03–5.93) | **0.000*** | 3.17 (1.68–5.98) | **0.000**** |
|  | ≥ 4 | 23 (65.7) | 12 (34.3) | 1.97 (0.88–4.39) | 0.097 | 2.67 (1.08–6.57) | 0.032** |
| Cohabitation with pets (dogs and/or cats) | No | 119 (73.5) | 43 (26.5) | 1 | – | 1 | – |
|  | Yes | 82 (59.4) | 56 (40.6) | 1.89 (1.16–3.08) | **0.010*** | 2.02 (1.13–3.60) | **0.018**** |
| Education level ^a^ | Secondary/ higher | 41 (71.9) | 16 (28.1) | 1 | – | 1 | – |
|  | Basic complete | 53 (56.4) | 41 (43.6) | 1.98 (0.98–4.02) | 0.057 | 1.63 (0.77–3.44) | 0.196 |
|  | Basic incomplete | 63 (76.8) | 19 (23.2) | 0.77 (0.36–1.67) | 0.513 | 0.67 (0.28–1.59) | 0.362 |
|  | Illiterate | 9 (75.0) | 3 (25.0) | 0.85 (0.20–3.56) | 0.829 | 0.68 (0.14–3.28) | 0.634 |
| Age | ≥60 | 26 (76.5) | 8 (23.5) | 1 | – | 1 | – |
|  | 45–59 | 40 (74.1) | 14 (25.9) | 1.13 (0.42–3.09) | 0.801 | 0.92 (0.32–2.71) | 0.889 |
|  | 30–44 | 62 (61.4) | 39 (38.6) | 2.04 (0.84–4.97) | 0.115 | 1.66 (0.62–4.47) | 0.317 |
|  | 15–29 | 38 (67.9) | 18 (32.1) | 1.54 (0.58–4.06) | 0.384 | 1.02 (0.33–3.18) | 0.965 |
|  | 0–14 | 35 (63.6) | 20 (36.4) | 1.86 (0.71–4.87) | 0.209 | – | – |

^a^ Only patients above the age of 15 were included in the analysis for this parameter

* p<0.05 in bivariate analysis

** p<0.05 in multivariate analysis

**Table 3.** Bivariate analysis of association between selected risk factors and *Shigella* spp. prevalence among study participants.

| **Variables** | **Categories** | ***Shigella* spp.** | | | **Univariate logistic analysis** | |
| --- | --- | --- | --- | --- | --- | --- |
|  |  | **N. tested** | **N. positive** | **%**  **prevalence** | **COR (95% CI)** | **p value** |
| Sex | Female | 194 | 28 | 14.4 | 1 | – |
|  | Male | 106 | 17 | 16.0 | 1.13 (0.59–2.18) | 0.710 |
| Age | ≥60 | 34 | 2 | 5.9 | 1 | – |
|  | 45–59 | 54 | 6 | 11.1 | 2.00 (0.38–10.52) | 0.414 |
|  | 30–44 | 101 | 24 | 23.8 | 4.98 (1.11–22.33) | **0.036*** |
|  | 15–29 | 56 | 4 | 7.1 | 1.23 (0.21–7.10) | 0.817 |
|  | 0–14 | 55 | 9 | 16.4 | 3.13 (0.63–15.44) | 0.162 |
| Residence | Urban/Under urbanization | 22 | 2 | 9.1 | 1 | – |
|  | Suburban/Peri-urban | 278 | 43 | 15.5 | 1.83 (0.41–8.11) | 0.427 |
| Education level ^a^ | Secondary/ higher | 57 | 6 | 10.5 | 1 | – |
|  | Basic complete | 94 | 22 | 23.4 | 2.60 (0.98–6.86) | 0.054 |
|  | Basic incomplete | 82 | 7 | 8.5 | 0.79 (0.25–2.50) | 0.692 |
|  | Illiterate | 12 | 1 | 8.3 | 0.77 (0.08–7.08) | 0.820 |
| Occupation ^a^ | Employed | 121 | 14 | 11.6 | 1 | – |
|  | Unemployed | 124 | 22 | 17.7 | 1.64 (0.80–3.40) | 0.175 |
| Consumption of chicken | No | 95 | 10 | 10.5 | 1 | – |
|  | Yes | 205 | 35 | 17.1 | 1.75 (0.83–3.70) | 0.144 |
| Consumption of eggs | No | 143 | 23 | 16.1 | 1 | – |
|  | Yes | 157 | 22 | 14.0 | 0.85 (0.45–1.60) | 0.616 |
| Consumption of raw vegetables | No | 164 | 22 | 13.4 | 1 | – |
|  | Yes | 136 | 23 | 16.9 | 1.31 (0.70–2.48) | 0.399 |
| Source of drinking water | Tap | 254 | 36 | 14.2 | 1 | – |
|  | Public standpipe/well | 25 | 5 | 20.0 | 1.51 (0.53–4.29) | 0.435 |
|  | Mineral and/or tap | 21 | 4 | 19.1 | 1.43 (0.45–4.48) | 0.544 |
| Boiling of drinking water | Yes | 75 | 14 | 18.7 | 1 | – |
|  | No | 225 | 31 | 13.8 | 0.70 (0.35–1.39) | 0.306 |
| Use of latrine^a,b^ | No | 73 | 9 | 12.3 | 1 | – |
|  | Yes | 172 | 27 | 15.7 | 1.32 (0.59–2.98) | 0.497 |
| Cohabitation with pets (dogs and/or cats) | No | 162 | 22 | 13.6 | 1 | – |
|  | Yes | 138 | 23 | 16.7 | 1.27 (0.67–2.40) | 0.456 |
| Cohabitation with chickens and/or ducks | No | 215 | 28 | 13.0 | 1 | – |
|  | Yes | 85 | 17 | 20.0 | 1.67 (0.86–3.24) | 0.130 |
| HIV | No | 150 | 16 | 10.7 | 1 | – |
|  | Yes | 150 | 29 | 19.3 | 2.01 (1.04–3.88) | **0.038*** |
| Viral load range | <1,000 | 128 | 24 | 18.7 | 1 | – |
|  | >1,000 | 20 | 5 | 25.0 | 1.44 (0.48–4.36) | 0.514 |
| Severity of symptoms | 0–1 | 148 | 24 | 16.2 | 1 | – |
|  | 2–3 | 117 | 18 | 15.4 | 0.94 (0.48–1.83) | 0.854 |
|  | ≥ 4 | 35 | 3 | 8.6 | 0.48 (0.14–1.71) | 0.260 |
| BMI | Normal | 204 | 31 | 15.2 | 1 | – |
|  | Underweight | 27 | 2 | 7.4 | 0.45 (0.10–1.98) | 0.289 |
|  | Overweight | 69 | 12 | 17.4 | 1.17 (0.57–2.44) | 0.666 |

^a^ Only patients above the age of 15 were included in the analysis for this parameter

^b^ instead of toilet

* p < 0.05 in bivariate analysis

**Table 4.** Multivariate logistic regression analysis of potential risk factors and their association with prevalence of *Shigella* spp. among study participants.

| **Variables** | **Categories** | ***Shigella* spp.** | | **COR (95%CI)** | **p–value** | **AOR (95%CI)** | **p–value** |
| --- | --- | --- | --- | --- | --- | --- | --- |
|  |  | **Negative (%)** | **Positive (%)** |  |  |  |  |
| Age | ≥60 | 32 (94.1) | 2 (5.9) | 1 | – | 1 | – |
|  | 45–59 | 48 (88.9) | 6 (11.1) | 2.00 (0.38–10.52) | 0.414 | 1.27 (0.21–7.58) | 0.790 |
|  | 30–44 | 77 (76.2) | 24 (23.8) | 4.98 (1.11–22.33) | **0.036*** | 2.63 (0.52–13.38) | 0.242 |
|  | 15–29 | 52 (92.9) | 4 (7.1) | 1.23 (0.21–7.10) | 0.817 | 0.56 (0.09–3.67) | 0.550 |
|  | 0–14 | 46 (83.6) | 9 (16.4) | 3.13 (0.63–15.44) | 0.162 | – | – |
| HIV | No | 134 (89.3) | 16 (10.7) | 1 | – | 1 | – |
|  | Yes | 121 (80.7) | 29 (19.3) | 2.01 (1.04–3.88) | **0.038*** | 1.68 (0.62–4.55) | 0.307 |
| Education level ^a^ | Secondary/ higher | 51 (89.5) | 6 (10.5) | 1 | – | 1 | – |
|  | Basic complete | 72 (76.6) | 22 (23.4) | 2.60 (0.98–6.86) | 0.054 | 2.40 (0.85–6.79) | 0.100 |
|  | Basic incomplete | 75 (91.5) | 7 (8.5) | 0.79 (0.25–2.50) | 0.692 | 0.67 (0.20–2.25) | 0.515 |
|  | Illiterate | 11 (91.7) | 1 (8.3) | 0.77 (0.08–7.08) | 0.820 | 0.91 (0.09–9.39) | 0.934 |
| Cohabitation with chickens and/or ducks | No | 187 (87.0) | 28 (13.0) | 1 | – | 1 | – |
|  | Yes | 68 (80.0) | 17 (20.0) | 1.67 (0.86–3.24) | 0.130 | 1.52 (0.65–3.54) | 0.331 |
| Consumption of chicken | No | 85 (89.5) | 10 (10.5) | 1 | – | 1 | – |
|  | Yes | 170 (82.9) | 35 (17.1) | 1.75 (0.83–3.70) | 0.144 | 2.04 (0.84–4.95) | 0.115 |
| Occupation ^a^ | Employed | 107 (88.4) | 14 (11.6) | 1 | – | 1 | – |
|  | Unemployed | 102 (82.3) | 22 (17.7) | 1.64 (0.80–3.40) | 0.175 | 2.03 (0.91–4.52) | 0.083 |

^a^ Only patients above the age of 15 were included in the analysis for this parameter

* p < 0.05 in bivariate analysis

**Table 5.** Bivariate analysis of association between selected risk factors and *Campylobacter* spp. prevalence among study participants.

| **Variables** | **Categories** | ***Campylobacter* spp.** | | | **Univariate logistic analysis** | |
| --- | --- | --- | --- | --- | --- | --- |
|  |  | **N. tested** | **N. positive** | **%prevalence** | **COR (95% CI)** | **p value** |
| Sex | Female | 194 | 10 | 5.2 | 1 | – |
|  | Male | 106 | 3 | 2.8 | 0.54 (0.14–1.99) | 0.352 |
| Age | ≥60 | 34 | 1 | 2.9 | 1 | – |
|  | 45–59 | 54 | 2 | 3.7 | 1.27 (0.11–14.51) | 0.849 |
|  | 30–44 | 101 | 4 | 3.9 | 1.36 (0.15–12.57) | 0.787 |
|  | 15–29 | 56 | 3 | 5.4 | 1.86 (0.19–18.65) | 0.596 |
|  | 0–14 | 55 | 3 | 5.5 | 1.90 (0.19–19.02) | 0.585 |
| Residence | Urban/Under urbanization | 22 | 1 | 4.5 | 1 | – |
|  | Suburban/Peri-urban | 278 | 12 | 4.3 | 0.95 (0.12–7.63) | 0.959 |
| Education level ^a^ | Secondary/ higher | 57 | 0 | 0.0 | – † | – |
|  | Basic complete | 94 | 6 | 6.4 | – | – |
|  | Basic incomplete | 82 | 3 | 3.7 | – | – |
|  | Illiterate | 12 | 1 | 8.3 | – | – |
| Occupation ^a^ | Employed | 121 | 3 | 2.5 | 1 | – |
|  | Unemployed | 124 | 7 | 5.7 | 2.35 (0.59–9.32) | 0.223 |
| Consumption of chicken | No | 95 | 4 | 4.2 | 1 | – |
|  | Yes | 205 | 9 | 4.4 | 1.04 (0.31–3.48) | 0.944 |
| Consumption of eggs | No | 143 | 4 | 2.8 | 1 | – |
|  | Yes | 157 | 9 | 5.7 | 2.11 (0.64–7.02) | 0.222 |
| Consumption of raw vegetables | No | 164 | 7 | 4.3 | 1 | – |
|  | Yes | 136 | 6 | 4.4 | 1.04 (0.34–3.16) | 0.952 |
| Source of drinking water | Tap | 254 | 10 | 3.9 | 1 | – |
|  | Public standpipe/well | 25 | 2 | 8.0 | 2.12 (0.44–10.27) | 0.350 |
|  | Mineral and/or tap | 21 | 1 | 4.8 | 1.22 (0.15–10.02) | 0.853 |
| Boiling of drinking water | Yes | 75 | 5 | 6.7 | 1 | – |
|  | No | 225 | 8 | 3.6 | 0.52 (0.16–1.63) | 0.259 |
| Use of latrine^a,b^ | No | 108 | 4 | 5.5 | 1 | – |
|  | Yes | 192 | 6 | 3.5 | 0.62 (0.17–2.28) | 0.474 |
| Cohabitation with pets (dogs and/or cats) | No | 162 | 5 | 3.1 | 1 | – |
|  | Yes | 138 | 8 | 5.8 | 1.93 (0.62–6.05) | 0.258 |
| Cohabitation with chickens and/or ducks | No | 215 | 8 | 3.7 | 1 | – |
|  | Yes | 85 | 5 | 5.9 | 1.62 (0.51–5.09) | 0.410 |
| HIV | No | 150 | 6 | 4.0 | 1 | – |
|  | Yes | 150 | 7 | 4.7 | 1.17 (0.39 – 3.58) | 0.777 |
| Viral load range | <1,000 | 128 | 6 | 4.7 | 1 | – |
|  | >1,000 | 20 | 1 | 5.0 | 1.07 (0.12–9.39) | 0.950 |
| Severity of symptoms | 0–1 | 148 | 5 | 3.4 | 1 | – |
|  | 2–3 | 117 | 4 | 3.4 | 1.01 (0.27–3.86) | 0.986 |
|  | ≥ 4 | 35 | 4 | 11.4 | 3.69 (0.94–14.54) | 0.062 |
| BMI | Normal | 204 | 9 | 4.4 | 1 | – |
|  | Underweight | 27 | 2 | 7.4 | 1.73 (0.35–8.48) | 0.497 |
|  | Overweight | 69 | 2 | 2.9 | 0.65 (0.14–3.07) | 0.583 |

^a^ Only patients above the age of 15 were included in the analysis for this parameter

^b^ instead of toilet

**†** Education level was not analyzed in logistic regression given that *Campylobacter* spp. was not found in one of the outcomes.

**Table 6.** Multivariate logistic regression analysis of potential risk factors and their association with prevalence of *Campylobacter* spp. among study participants.

| **Variables** | **Categories** | ***Campylobacter* spp.** | | **COR (95%CI)** | **p–value** | **AOR (95%CI)** | **p–value** |
| --- | --- | --- | --- | --- | --- | --- | --- |
|  |  | **Negative (%)** | **Positive (%)** |  |  |  |  |
| Severity of symptoms | 0–1 | 143 (96.6) | 5 (3.4) | 1 | – | 1 | – |
|  | 2–3 | 113 (96.6) | 4 (3.4) | 1.01 (0.27–3.86) | 0.986 | 0.62 (0.11–3.49) | 0.589 |
|  | ≥ 4 | 31 (88.6) | 4 (11.4) | 3.69 (0.94–14.54) | 0.062 | 4.33 (0.95–19.72) | 0.059 |
| Occupation^a^ | Employed | 118 (97.5) | 3 (2.5) | 1 | – | 1 | – |
|  | Unemployed | 117 (94.4) | 7 (5.6) | 2.35 (0.59–9.32) | 0.223 | 1.68 (0.40–7.09) | 0.483 |
| Consumption of eggs | No | 139 (97.2) | 4 (2.8) | 1 | – | 1 | – |
|  | Yes | 148 (94.3) | 9 (5.7) | 2.11 (0.64–7.02) | 0.222 | 1.63 (0.43–6.21) | 0.471 |

^a^ Only patients above the age of 15 were included in the analysis for this parameter
